# Transient Congenital Nephrotic Syndrome in Neonates: Two Case Reports and Review of Recent Literature

**DOI:** 10.7759/cureus.94722

**Published:** 2025-10-16

**Authors:** Tahani Almohayya, Abdulrahman A Al Zahrani, Samya M Edris, Hammad Alshaya

**Affiliations:** 1 Department of Neonatal Intensive Care Unit, Dr. Sulaiman Al Habib Medical Group, Riyadh, SAU; 2 Department of Pediatrics, Dr. Sulaiman Al Habib Medical Group, Riyadh, SAU

**Keywords:** conginatal nephrotic syndrome, hydrops fetalis, pleural effusion, proteinuria, transient conenital nephrotic syndrome

## Abstract

We report two cases of non-genetic transient congenital nephrotic syndrome (CNS) in neonates, both presenting with hydrops fetalis associated with nephrotic range proteinuria and significant hypoalbuminemia. Investigations did not reveal any underlying genetic cause or congenital infection to explain the nephrotic syndrome. Both cases received supportive therapy and showed spontaneous recovery with resolution of proteinuria and normalization of serum albumin in a few weeks. These cases underscore the diagnostic and prognostic uncertainties in CNS without identifiable genetic or infectious causes and highlight a possible transient or stress-induced phenotype not previously well-characterized in the literature. Furthermore, this raises the question of whether we need to redefine the definition of CNS by adding a minimum duration after which an infant will be labeled as having CNS.

## Introduction

Congenital nephrotic syndrome (CNS) is a rare kidney condition marked by heavy proteinuria, hypoalbuminemia, and edema presenting during the first three months of life. In most cases, there is a genetic defect in the components of the glomerular filtration barrier, especially nephrin and podocin [1,2]. Mutations in five different genes (NPHS1, NPHS2, PLCE1, WT1, and LAMB2) are responsible for most of the cases. CNS can also be acquired after intrauterine or perinatal infections [3].

The management is mainly supportive, involving control of edema, a high-calorie diet, thyroid hormone replacement, mineral supplementation, prevention of thrombotic events, and immediate handling of infections. The outcome of CNS patients without major extra-renal manifestations after kidney transplantation is comparable with that of other children with chronic kidney disease.

Hydrops fetalis is a condition where fluid collection occurs in two or more compartments in the newborn. There are multiple possible underlying causes for this condition, ranging from liver and kidney problems to idiopathic and malnutrition-related issues. The underlying pathophysiology could be due to immune or non-immune causes. Immune causes are typically due to maternofetal alloimmunization, while non-immune causes stem from infection and genetic mutations [4].

The coexistence of these two conditions in a single neonate significantly complicates the clinical picture, with prognosis varying widely based on the neonatal age and initial need for resuscitation. Most cases of CNS progress rapidly to end-stage renal disease (ESRD), while only a few cases demonstrate improvement without the need for renal transplantation [5].

Here, we present two complex cases of newborns that experienced both conditions, notable for their atypical clinical course, extensive management, and eventual spontaneous resolution of proteinuria, hypoalbuminemia, and edema.

## Case presentation

Case 1

**Figure 1:**
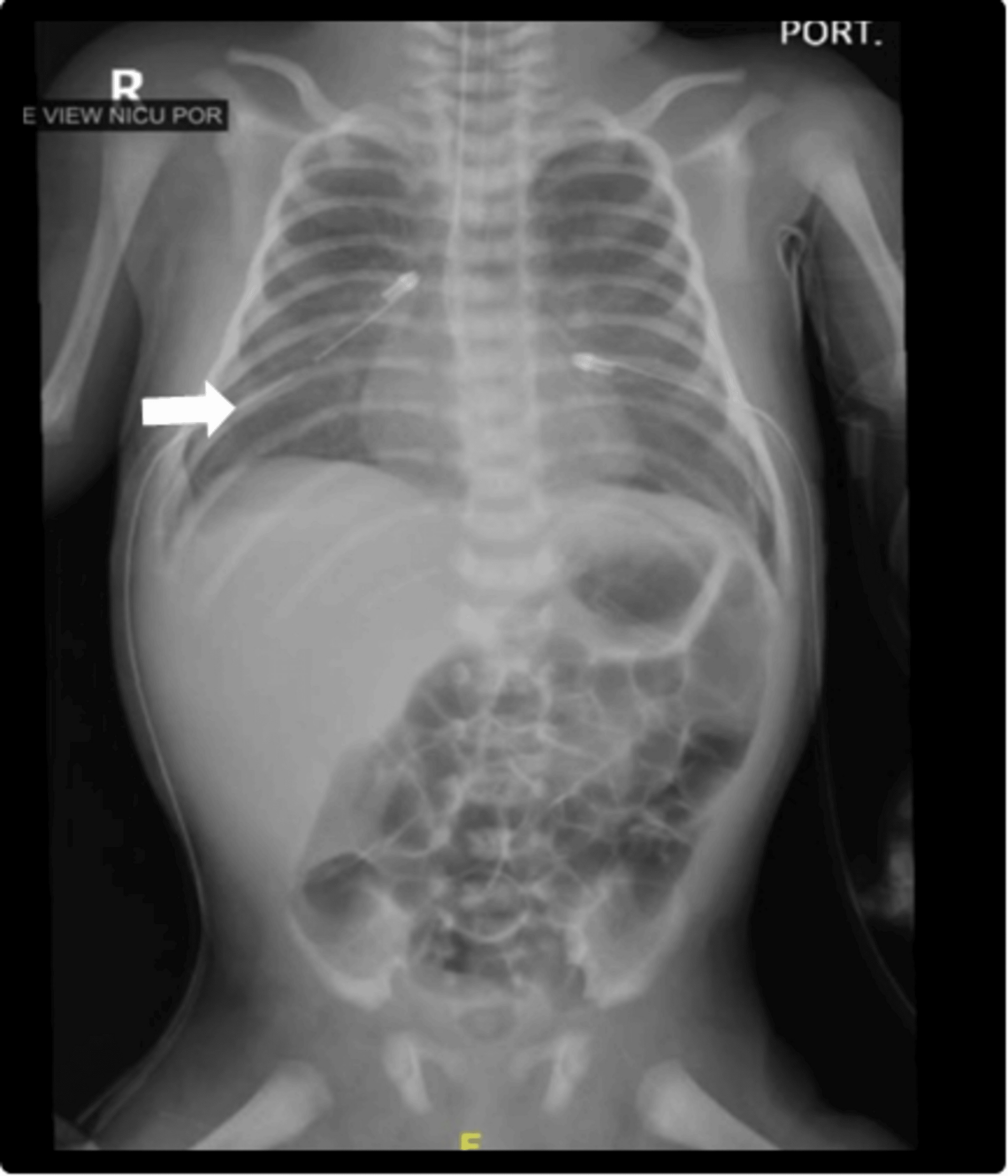
Chest and abdominal X-ray of a neonate with congenital nephrotic syndrome with bilateral pleural effusion that improved upon insertion of chest tubes (white arrow).

**Figure 2:**
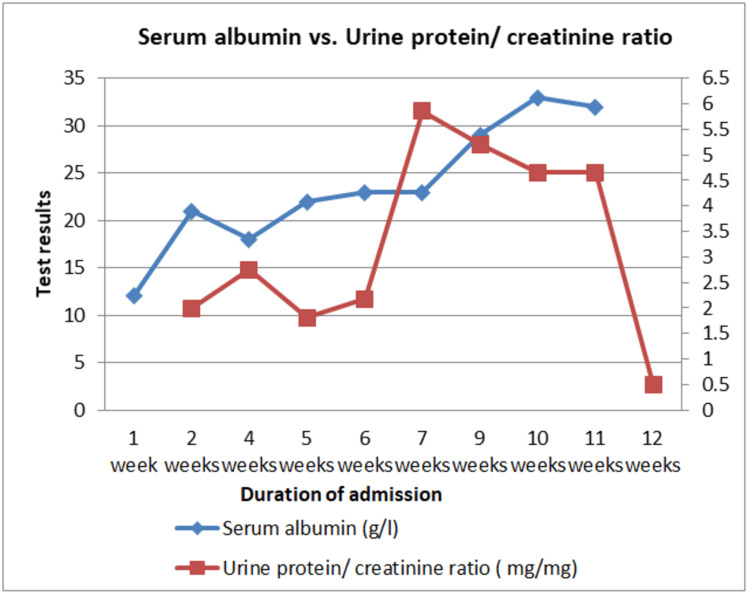
Time series graph showing serum albumin and urine protein/creatinine ratio results of a neonate with congenital nephrotic syndrome across time. The first line (red) shows serial results (left side) of the protein/creatinine ratio (mg/mg), while the second line (blue) shows the serial results of serum albumin (g/L) ( right side).

A 35-week preterm male neonate was diagnosed antenatally with hydrops fetalis with severe bilateral pleural effusions. At birth, he required resuscitation with intubation and mechanical ventilation. Examination revealed a severely distressed and edematous neonate with no apparent dysmorphic features. Initial laboratory investigations revealed low serum albumin (17 g/L) with nephrotic-range proteinuria (5.86 mg/mg), confirming the diagnosis of CNS. Abdominal ultrasound revealed significant ascites that necessitated paracentesis. Ascitic fluid analysis showed a transudative profile. This favored a non-infectious etiology of hydrops fetalis; however, no definite cause for the condition could be established. The respiratory distress worsened due to fluid collection in the chest (Figure 1), necessitating chest tube insertion and mechanical ventilation. In addition, no congenital infections were detected. The urine protein/creatinine ratio started improving by seven to eight weeks and normalized at the end of the admission period (Figure 2). Low serum albumin levels also resolved by the time of discharge (Figure 2), although repeated infusions were required during hospitalization. Whole exome sequencing was unremarkable. The infant was discharged home after 12 weeks of admission in good general health, with no evidence of edema. His last serum albumin was 33 g/L, and urine analysis showed no proteinuria. He was subsequently reviewed in the outpatient department at 14 weeks of age, where he remained clinically well through recent follow-up.

Case 2

**Figure 3:**
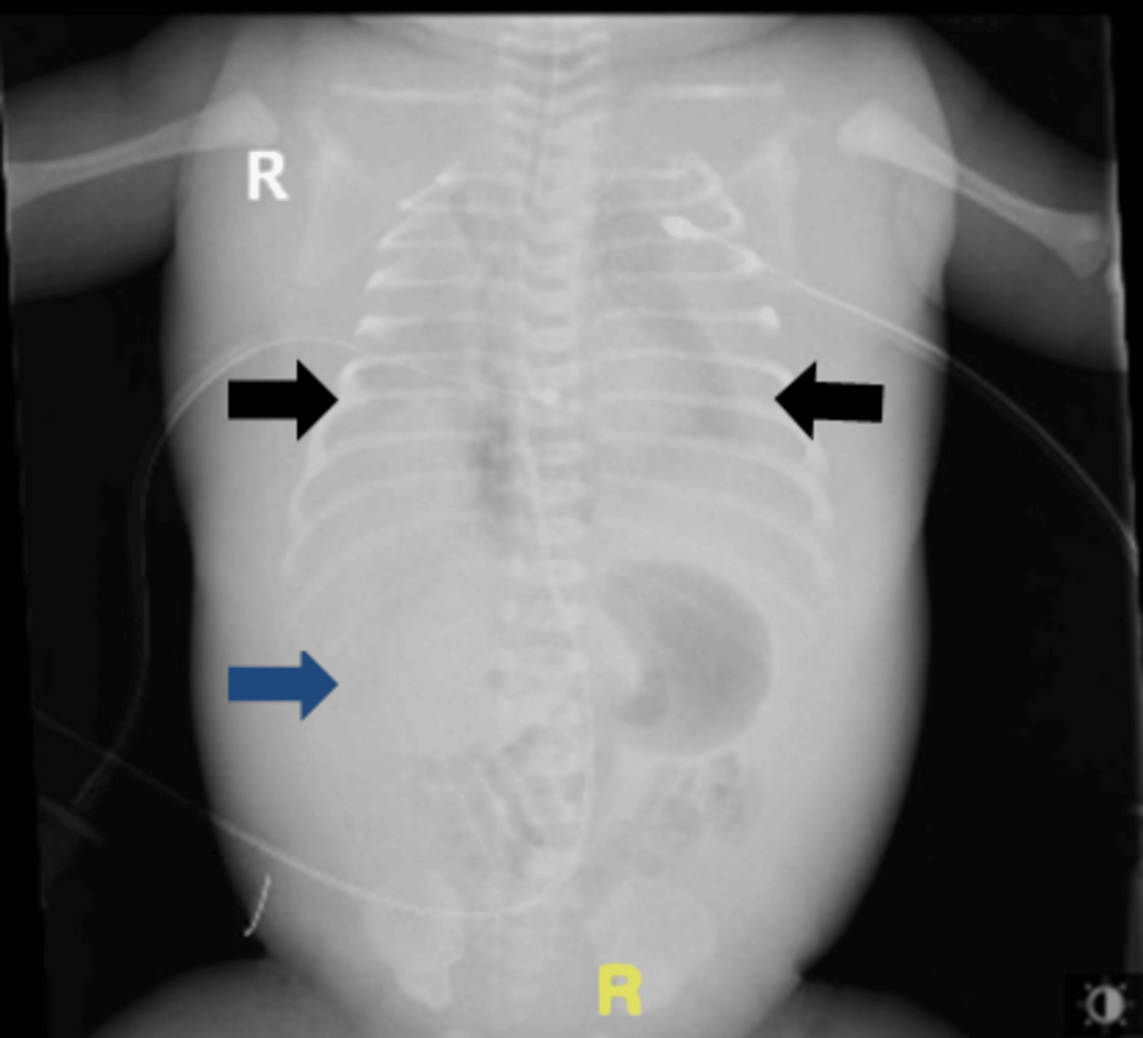
Abdominal and chest X-ray of a neonate with congenital nephrotic syndrome showing bilateral pleural effusion (black arrows) and abdominal ascites (blue arrow).

**Figure 4:**
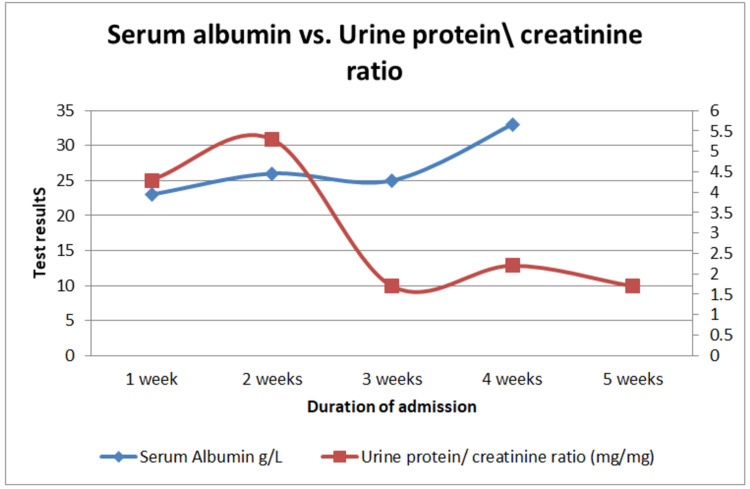
Time series graph showing serum albumin and urine protein/creatinine ratio results of a term neonate with congenital nephrotic syndrome across time in the first four weeks of life. The first line (red) shows serial results of the protein/creatinine ratio in mg/mg (left side), while the second line (blue) shows the serial result of serum albumin in g/L (right side).

A 37-week male neonate was diagnosed antenatally with an isolated right-sided pleural effusion. At birth, he required initial resuscitation and intubation with mechanical ventilation. Examination revealed a respiratory-distressed and edematous neonate with no apparent dysmorphic features and significant bilateral pleural effusions, which were subsequently drained via chest tubes (Figure 3). Initial laboratory investigations showed profound hypoalbuminemia (15 g/L) and nephrotic-range proteinuria (urine protein-to-creatinine ratio of 4 mg/mg), prompting suspicion of CNS. The infant also required multiple albumin infusions. Whole exome sequencing and congenital infectious workup were negative. Both the serum albumin level and urine protein/creatinine ratio dramatically improved over time (Figure 4). He was discharged at six weeks of age with no edema, a urine protein/creatinine ratio of 1.7 mg/mg, and a serum albumin of 41 g/L. Six weeks later, he was readmitted with a urinary tract infection, during which his albumin level was within the normal range at 34 g/L. The condition eventually resolved, and he was discharged again in good condition.

## Discussion

CNS is characterized by severe proteinuria, hypoalbuminemia, and edema. It typically carries a poor prognosis without the aid of renal replacement therapy. It is frequently caused by mutations in genes, such as NPHS1, NPHS2, PLCE1, WT1, and LAMB2, and less commonly by intrauterine infections. However, the cases presented here defy this conventional understanding.

We report two cases, one preterm and one term neonate, both presenting with features of CNS and hydrops fetalis, who demonstrated spontaneous normalization of serum albumin levels and urine protein-to-creatinine ratio within a few weeks of life. While some features of these cases differ, notably the longer admission duration and more complicated clinical course in the first patient, several similarities exist between the two. Both presented with hydrops fetalis and severe nephrotic features, with no identifiable genetic or infectious cause, and achieved full recovery without renal replacement therapy. Both infants were born to healthy mothers with otherwise uneventful pregnancies, aside from prenatal ultrasound findings, and both received high-intensity supportive NICU care. Renal biopsy was not performed, as it is generally not indicated unless persistent disease warrants evaluation before renal transplantation [6].

Spontaneous remission of CNS is exceedingly rare. Most reported cases progress to ESRD (if left untreated) and necessitate renal replacement therapy or transplantation [7]. Our case report suggests the presence of a non-genetic, self-limiting CNS subset, possibly triggered by perinatal insults such as sepsis, hemodynamic instability, or metabolic stress.

However, the absence of known genetic mutations in both cases may reflect the limitations of whole exome sequencing (WES), which cannot detect all genomic anomalies, such as intronic mutations, epigenetic alterations, or structural variants [8]. Whole genome sequencing (WGS), although currently expensive and less accessible, may offer greater diagnostic clarity in similar future cases.

A review of literature published in the last five years revealed similar case reports of non-classical CNS. One such report described a rare variant, CNS of the Finnish type (CNS-FT), which results in massive proteinuria, hypoproteinemia, edema, and hypercholesterolemia. Similar to our cases, symptoms in this subtype appear in the early days of life. A case with a similar presentation was reported from Saudi Arabia in 2020. However, unlike our current cases, it identified a specific mutation in the NPHS1 gene. In addition, that child was not distressed at birth and was diagnosed two months post-birth, potentially explaining the difference in early neonatal presentation. Furthermore, the improvement in the aforementioned case took several years to occur, in contrast to the sudden improvement observed in our cases after a few weeks [9].

Similarly, a case of a two-day-old female neonate was reported by Aydin et al. in 2023, presenting with edema, proteinuria, and hypoalbuminemia, and was subsequently diagnosed with CNS. After exclusion of secondary causes, genetic testing revealed a frameshift mutation in the NPHS1 gene. Remarkably, both edema and proteinuria gradually resolved with supportive management alone, without the need for renal replacement therapy [10].

Another case of spontaneous remission was reported in an 11-day-old baby who presented to the hospital with seizure and edema, which was later diagnosed as CNS due to the finding of proteinuria and low serum albumin. Contrary to our cases, however, this child did not need to have albumin infusion, and by the time he reached six months of age, the albumin started to increase until it normalized 18 months later. Another distinction in this case is the finding of a genetic mutation in NPHS1, which could explain the benign course in this particular case [11]. The normalization of renal tests (urinary protein to creatinine) could take more time, as in the case reported by Le et al. (2021), where the normalization took place at two years old in a girl who presented with abdominal distension and edema, which was later found to be CNS [12] (Table 1).

**Table 1:** Summary of recent similar cases of congenital nephrotic syndrome with spontaneous resolution.

Authors	Age of diagnosis	Presentation	Investigations	Genetic mutation	Outcome	Others
Aydin et al. (2023)[10]	2 days	Proteinuria, hypoalbuminemia, and edema	Serum albumin: 1.3 g/L, serum creatinine: 0.24 mg/dl (21.2 µmol/L), urinary protein/creatinine ratio: 41 mg/mg	NPHS1 gene	Initial improvement in one week and normalization after four months	Repeated weekly Albumin infusions were required
Espino et al. (2022)[11]	11-day term	Seizures and edema	Serum albumin: 0.5 g/L, total proteins: 32 g/L, serum creatinine: 13.26 μmol/L, with a urinary protein/creatinine ratio of 36.1 mg/mg	NPHS1 gene	Improvement after six months and normalization at 18 months	No albumin infusions were required
AlHassan et al. (2020) [9]	2 month	Proteinuria, hypoalbuminemia, and edema	Serum albumin: 22 g/l, total protein: 48 g/l	NPHS1 gene	Normalization at 16 months of age	Repeated albumin infusion
LI et al. (2021) [12]	1 month and 20 days	Abdominal distension and palpebral edema, proteinuria, and hypoalbuminemia	Protein-to-creatinine ratio: 28.49 mg\mg, albumin level: 10 g/L	NPHS1 gene	Normalization at two years	Albumin and frusemide injections
Sinha et al. (2020) (case series) [13]	42 days	Microcephaly, seizure, and sepsis	Serum albumin: 1.2 g/l, serum creatinine: 35 µmol/L, torch positive in only 12 cases	NPHS1 and PLCE-1 genes	Complete remission: n = 1, partial remission: n = 8)	Albumin infusion and angiotensin-converting enzyme inhibitors
Current first case	birth	Dyspnea, edema, proteinuria, and hypoalbuminemia	Serum albumin: 23 g/l, protein/creatinine ratio: 22 mg/mg	None	Normalization at two months	Received multiple albumin and furosemide injection
Current second case	birth	Respiratory distress, proteinuria, and hypoalbuminemia	Serum albumin: 15 g/L, protein-to-creatinine ratios: 4 mg/mg	None	Normalization after four weeks	Received multiple albumin injection

Albumin infusion and renal replacement therapy are typically required for CNS patients to decrease mortality from ESRD. Rarely, the illness can show signs of spontaneous remission. However, in a retrospective study of 49 patients with CNS (diagnosed between zero and three months), some of whom did not undergo genetic testing due to high cost, eight patients spontaneously improved without the need for renal replacement therapy, sharing a similar picture with our current two cases. Furthermore, while albumin infusion is an important part of the therapy, almost 64% of children who required renal transplant therapy in this study had already received steady albumin transfusions, making albumin infusion unlikely to be the sole cause of spontaneous remission in such cases [14].

A similar pattern was observed in a previous case series from India by Sinha et al. (2020) involving 65 children, of whom 45% were preterm. Only 23% of children in this cohort had undergone genetic testing, which revealed mutations in three associated genes: NPHS1, WT1, and PLCE-1. It is worth noting that only eight children in this study showed partial response, while only one showed complete response [13], confirming the overall poor prognosis of the disease.

In this paper, we explored unusual presentations of CNS that required intensive management, suggesting the involvement of distinct underlying causes and pathophysiological mechanisms in this rapidly resolving form of the disease. In addition, the transient nature observed in some of these cases highlights the need to reconsider the current diagnostic timeframe for CNS (birth to three months) and to potentially adopt a more clinically appropriate duration.

## Conclusions

The two cases highlighted in this study demonstrate a relatively rare, transient form of non-genetic/non-infectious congenital nephrotic syndrome. These cases presented with severe nephrotic features and hydrops fetalis, which resolved spontaneously. This outcome challenges the typical prognosis of CNS and suggests a possible self-limiting subtype, potentially triggered by perinatal stress. Further case reports and broader genetic studies are needed to improve diagnosis, predict outcomes, and guide the management of such atypical presentations.

## References

[1] Hölttä T, Jalanko H (2020). Congenital nephrotic syndrome: is early aggressive treatment needed? Yes. Pediatr Nephrol.

[2] Lee BH, Ahn YH, Choi HJ (2009). Two Korean infants with genetically confirmed congenital nephrotic syndrome of Finnish type. J Korean Med Sci.

[3] Anderson S (2022). Congenital nephrotic syndrome of the finnish type in a dominican newborn: an overview and case report. Neonatal Netw.

[4] Younge T, Ottolini KM, Al-Kouatly HB, Berger SI (2023). Hydrops fetalis: incidence, etiologies, management strategies, and outcomes. Res Rep Neonatol.

[5] Boyer O, Schaefer F, Haffner D (2021). Management of congenital nephrotic syndrome: consensus recommendations of the ERKNet-ESPN Working Group. Nat Rev Nephrol.

[6] Schnuelle P (2023). Renal biopsy for diagnosis in kidney disease: indication, technique, and safety. J Clin Med.

[7] Holmberg C, Jalanko H (2014). Congenital nephrotic syndrome and recurrence of proteinuria after renal transplantation. Pediatr Nephrol.

[8] Burdick KJ, Cogan JD, Rives LC (2020). Limitations of exome sequencing in detecting rare and undiagnosed diseases. Am J Med Genet A.

[9] AlHassan A, AlKadhem SM, Alkhalifah F, Almajed JM, Alwabari ME (2020). Congenital nephrotic syndrome with a novel presentation in Saudi Arabia. Cureus.

[10] Aydin Z, İnözü M, Sahin I, Bayrakci U (2023). A case series of 4 patients with congenital nephrotic syndrome. Çocuk Derg J Child.

[11] Espinosa LG, Santoveña AZ, Blanco JN, Alvariño MG, Feito JB, Hijosa MM (2022). Spontaneous remission in a child with an NPHS1-based congenital nephrotic syndrome. Clin Kidney J.

[12] Li Z, Zhuang L, Han M, Li F (2021). A case report of congenital nephrotic syndrome caused by new mutations of NPHS1. J Int Med Res.

[13] Sinha R, Vasudevan A, Agarwal I (2020). Congenital nephrotic syndrome in India in the current era: a multicenter case series. Nephron.

[14] Constantinescu AR, Mattoo TK, Smoyer WE (2022). Clinical presentation and management of nephrotic syndrome in the first year of life: a report from the Pediatric Nephrology Research Consortium. Front Pediatr.

